# Targeting LAG3/GAL-3 to overcome immunosuppression and enhance anti-tumor immune responses in multiple myeloma

**DOI:** 10.1038/s41375-021-01301-6

**Published:** 2021-07-21

**Authors:** Jooeun Bae, Fabrizio Accardi, Teru Hideshima, Yu-Tzu Tai, Rao Prabhala, Aaron Shambley, Kenneth Wen, Sean Rowell, Paul G. Richardson, Nikhil C. Munshi, Kenneth C. Anderson

**Affiliations:** 1 Dana-Farber Cancer Institute, Boston, MA USA; 2 Harvard Medical School, Boston, MA USA

**Keywords:** Immunotherapy, Preclinical research, Cancer microenvironment

## Abstract

Immune profiling in patients with monoclonal gammopathy of undetermined significance (MGUS), smoldering multiple myeloma (SMM), and multiple myeloma (MM) provides the framework for developing novel immunotherapeutic strategies. Here, we demonstrate decreased CD4^+^ Th cells, increased Treg and G-type MDSC, and upregulation of immune checkpoints on effector/regulatory and CD138^+^ cells in MM patients, compared MGUS/SMM patients or healthy individuals. Among the checkpoints profiled, LAG3 was most highly expressed on proliferating CD4^+^ Th and CD8^+^ Tc cells in MM patients BMMC and PBMC. Treatment with antibody targeting LAG3 significantly enhanced T cells proliferation and activities against MM. XBP1/CD138/CS1-specific CTL generated in vitro displayed anti-MM activity, which was further enhanced following anti-LAG3 treatment, within the antigen-specific memory T cells. Treg and G-type MDSC weakly express LAG3 and were minimally impacted by anti-LAG3. CD138^+^ MM cells express GAL-3, a ligand for LAG3, and anti-GAL-3 treatment increased MM-specific responses, as observed for anti-LAG3. Finally, we demonstrate checkpoint inhibitor treatment evokes non-targeted checkpoints as a cause of resistance and propose combination therapeutic strategies to overcome this resistance. These studies identify and validate blockade of LAG3/GAL-3, alone or in combination with immune strategies including XBP1/CD138/CS1 multipeptide vaccination, to enhance anti-tumor responses and improve patient outcome in MM.

## Introduction

Despite major improvements in the treatment of multiple myeloma (MM), novel agents targeting the tumor and its microenvironment are urgently needed. The reciprocal interaction between MM and bone marrow (BM) accessory cells induces genomic, epigenomic, and functional changes, which in turn promote tumor development and progression, cell adhesion mediated-drug resistance, and immunosuppression. In prior studies, we and others have delineated mechanisms and sequelae of interactions among MM, stromal, and accessory cells [1–3]. These studies have enhanced our understanding of MM pathogenesis, delineated changes within tumor cells and the BM milieu underlying progression from monoclonal gammopathy of undetermined significance (MGUS) to smoldering MM (SMM) to active MM, and provided the framework for overcoming immunosuppression in the BM milieu.

Immunotherapeutic approaches to develop tumor-specific memory T cells and overcome inhibitory mechanisms in the tumor microenvironment have the potential to achieve prolonged anti-tumor immune and clinical responses. Currently, one of the most effective therapeutic strategies modulates immune checkpoints, which regulate the balance between immune response and tolerance [4, 5]. Targeting immune suppressive cells, blocking inhibitory molecules on suppressive/regulatory and tumor cells, and activating costimulatory molecules on effector cells also represent promising therapeutic approaches to enhance anti-tumor immunity and improve therapeutic efficacy. In MM, PD1 blockade in combination with either pomalidomide or lenalidomide was associated with adverse outcome, which has limited evaluation of other immune modulators (checkpoint inhibitors) in the clinic. A better understanding of the immunologic effects of checkpoint inhibitors and immune agonists in the BM tumor microenvironment would further delineate their role in pathogenesis and inform their therapeutic application, alone or in combination with other immunotherapies.

Cancer vaccines have been shown to generate antigen-specific effector T cells against tumor with a favorable therapeutic index [6–8]. Importantly, cancer vaccine therapies have the potential to develop memory CD8^+^ CTL specifically directed at selected tumor-associated antigens and induce long-term anti-tumor immunity [9, 10]. Although vaccines induce antigen-specific memory CD8^+^ CTL expressing costimulatory (CD28, 41BB) and activation (CD38, CD69) markers, these memory CTL also upregulate various inhibitory checkpoints (CTLA4, PD1, LAG3, TIM3, VISTA), which may in turn abrogate their function [11–13]. Previously, in preclinical and clinical studies, we reported that XBP1/CD138/CS1 multipeptide can induce antigen-specific memory CTL against MM [14–18], and that this therapeutic approach when combined with optimal immune modulators may further overcome immunosuppression characteristic of MM [19, 20].

In the current studies, we first investigated the potential impact of checkpoint inhibitors and immune agonists on effector T cells, accessory cells, and regulatory cells in MM. These studies demonstrate increased immune suppressor cells and upregulated inhibitory checkpoint molecules in patients with MM patients (newly diagnosed, relapsed, relapsed/refractory) compared to premalignant disease patient (MGUS, SMM). We show that blocking immune checkpoints (PD1, LAG3), alone and in combination, can enhance effector T-cell responses in the tumor microenvironment of MM patients to a greater extent than by stimulating costimulatory molecules (OX40, GITR). Importantly, we demonstrated increased LAG3 expression on proliferating CD3^+^ T cells in MM patient BMMC and PBMC, as well as robust surface and intracellular expression of its ligand, GAL-3, in CD138^+^ patient MM cells and MM cell lines. Moreover, LAG3/GAL-3 blockade can efficiently enhance the proliferation of T cells in MM patients and functional activities of MM-specific CTL, including XBP1/CD138/CS1-targeting memory CD8^+^ T cells, against MM. These studies provide the rationale for inhibiting LAG3/GAL-3, in combination with immunotherapy including a cancer vaccine, to overcome the immune suppressive tumor microenvironment, enhance anti-tumor-specific immune responses, and improve patient outcome in MM.

## Methods

### Cell lines and preparation of tumor cell lysates or irradiated whole tumor cells

MM cell lines U266, McCAR, HSB2, MM1S, RPMI8226, OPM2, H929, ANBL6, OCIMY5, AMO1, and KMS11 were obtained from ATCC (Manassas, VA). The T2 cell line, a human B- and T-cell hybrid expressing HLA-A2 molecules, was provided by Dr J. Molldrem (University of Texas M. D. Anderson Cancer Center, Houston, TX). The cell lines were cultured in DMEM media supplemented with 10% fetal calf serum (BioWhittaker, Walkersville, MD), 100 IU/ml penicillin, and 100 µg/ml streptomycin (Gibco-Life Technologies, Rockville, MD). A mixture of ten MM cell lines was utilized to prepare tumor cell lysates by repeated (10X) cycles of freeze (−140 °C)/thaw (37 °C) or prepared as irradiated (20 Gy) whole tumor cells as sources of MM antigen stimulation.

### Reagents

Fluorochrome-conjugated anti-human monoclonal antibodies (mAb) specific to CD3, CD4, CD8, CD11b, CD11b, CD14, CD15, CD25, CD28, CD33, CD38, CD69, CD138, FOXP3, HLA-DR, PD1, PD-L1, PD-L2, CTLA4, LAG3, TIM3, VISTA, ICOS, OX40, GITR, GAL-3, GAL-9, ICOS-L, HLA-DP/DQ/DR, CCR7, CD45RO, CD69, CD107a, or IFN-γ were purchased from Becton Dickinson (BD) (San Diego, CA), LifeSpan Biosciences (Seattle, WA), BioLegend (San Diego, CA), or eBioscience (San Diego, CA). Live/Dead Aqua Fixable Cell Stain Kit was purchased from Molecular Probes (Grand Island, NY). Recombinant human GM-CSF was obtained from Immunex (Seattle, WA), and human IL-2, IL-4, IFN-α, and TNF-α were purchased from R&D Systems (Minneapolis, MN). Clinical grade checkpoint inhibitor (anti-PD1, anti-LAG3) or immune agonist (anti-OX40, anti-GITR) were provided by Bristol Myers Squibb (New York, NY).

### BM or peripheral blood (PB) samples from MGUS, SMM, or MM patients or healthy donors

BM aspirates and PB samples were obtained from patients with MGUS [BM: *N* = 5, PB: *N* = 5] and SMM [BM: *N* = 5, PB: *N* = 5] and patients with MM (newly diagnosed [BM: *N* = 18, PB: *N* = 10], relapsed [BM: *N* = 14, PB: *N* = 12], relapsed/refractory [BM: *N* = 18, PB: *N* = 12]) after informed consent, in accordance with the Declaration of Helsinki, and with approval by the Institutional Review Board at Dana-Farber Cancer Institute (Boston, MA). In addition, healthy individuals BM [*N* = 5] or leukapheresis [*N* = 12] products were purchased from either AllCells (Alameda, CA) or the Blood Donor Center at Boston Children’s Hospital (Boston, MA), respectively. Mononuclear cells were isolated from BM (BMMC) or PB (PBMC) by standard density gradient centrifugation using Ficoll-Paque^TM^ Plus (Amersham Pharmacia Biotech AB, Uppsala Sweden) and used in these studies.

### Phenotypic characterization of immune and regulatory cell subsets and expression of checkpoints or costimulatory molecules

BMMC or PBMC from patients with MGUS, SMM, MM, or healthy individuals were evaluated by flow cytometry analyses by staining cells with fluorochrome-conjugated mAb specific to each cell surface antigen for 30 min at room temperature, followed by LIVE/DEAD reagent staining to confirm viability. Regulatory T cells (Treg) were identified by cell surface staining (CD3, CD4, CD8, CD25), permeabilized using Foxp3/Transcription Factor Staining Buffer Set (eBioscience) and stained for intracellular FOXP3 expression. Myeloid-derived suppressor cells (MDSC) were identified as G-type MDSC (CD11b^+^ CD33^+^ HLA-DR^low/−^ CD14^−^ CD15^+^) and M-type MDSC (CD11b^+^ CD33^+^ HLA-DR^low/−^ CD14^+^ CD15^−^). Cells were acquired using a BD Fortessa X-20 (BD Biosciences) flow cytometer, and the data were analyzed using DIVA™ v8.0 (BD) or FlowJo v10.0.7 (Tree star, Ashland, OR) software.

### Generation of MM-specific CTL ex vivo with immunogenic XBP1/CD138/CS1 peptides

XBP1/CD138/CS1-specific CTL were generated ex vivo after four cycles of weekly stimulation of HLA-A2^+^ CD3^+^ T lymphocytes (*N* = 5) with a cocktail of four peptides containing heteroclitic XBP1 US_184-192_ (YISPWILAV), heteroclitic XBP1 SP_367-375_ (YLFPQLISV), native CD138_260-268_ (GLVGLIFAV), and native CS1_239-247_ (SLFVLGLFL), as described previously [14–17].

### Cell proliferation by carboxy fluorescein succinimidyl ester (CFSE) tracking

Proliferation of specific cell populations was evaluated using CFSE-based proliferation assays (*N* = 5). In brief, MM patient BMMC, PBMC, or XBP1/CD138/CS1-specific CTL were labeled with CFSE (Molecular Probes, Eugene, OR) and incubated with clinical grade checkpoint inhibitor (1 μg/ml) or immune agonist (1 μg/ml) in the presence of low dose (20 units) IL-2, with or without stimulation with irradiated MM cells (patients’ cells, cell line) or MM lysates. After 4–7 days incubation, the cells were stained with LIVE/DEAD reagent and specific fluorochrome-conjugated mAbs, washed, fixed in 2% paraformaldehyde, and acquired using a LSRII Fortessa^TM^ flow cytometer.

### XBP1/CD138/CS1-specific CTL functional activities measured by CD107a degranulation and intracellular IFN-γ production

The anti-tumor activities of MM-specific CTL were measured by CD107a degranulation and IFN**-**γ production against MM (*N* = 5). In brief, XBP1/CD138/CS1-specific CTL were treated with clinical grade anti-PD1 or anti-LAG3 (1 μg/ml) for 24 h. The cells were then cultured with U266 MM cells in the presence of CD107a mAb. After 1-h incubation, brefeldin A (BD) and monensin (BD) were added, and cultures were incubated for an additional 5 h. Cells were harvested, washed in PBS, stained with LIVE/DEAD reagent, washed, incubated with fluorochrome-conjugated mAb to identify T cells, allowing for assays of their functional activity against MM. After surface staining, cells were fixed/permeabilized, stained for Th1 cytokines, washed with Perm/Wash solution (BD), and analyzed by flow cytometry. Multipeptide-CTL specific CD107a degranulation and Th1 cytokine production were analyzed using DIVA™ v8.0 or FlowJo v10.0.7 software.

### Statistical analysis

Results are presented as mean ± standard error. Groups were compared using an unpaired Student’s *t* test. Differences were considered significant when *p* < 0.05.

## Results

### Impact of clinical grade immune modulator treatment on proliferation of MM patient T cells expressing checkpoint or costimulatory molecules

**Fig. 1:**
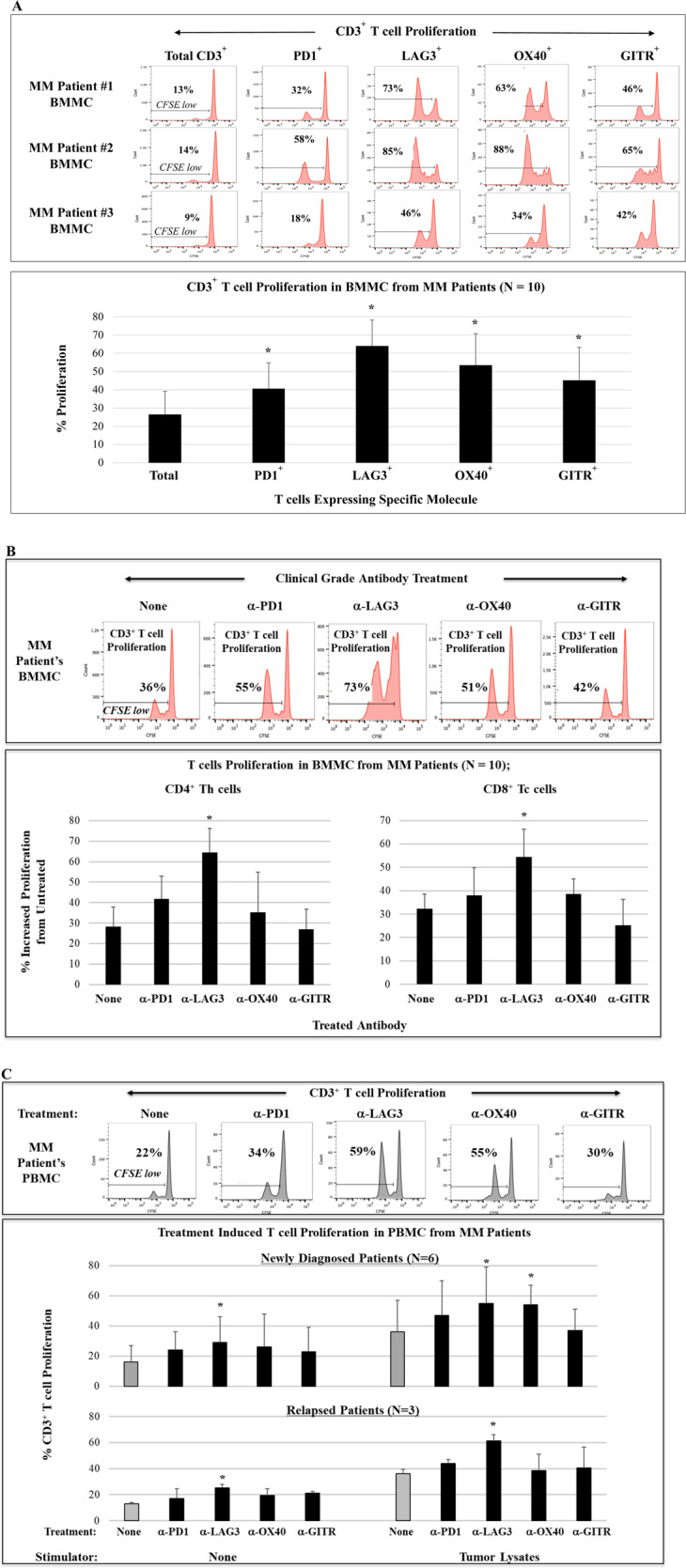
Characterization of checkpoint expression on MM patient T cells. BMMC from MM patients (newly diagnosed, relapsed, relapsed/refractory) were treated with low dose (20 units) IL-2 and evaluated for proliferation of specific T-cell subsets in CFSE assay. **A** CD3^+^ T cells expressing PD1, LAG3, OX40, or GITR had significantly (*p < 0.05) higher proliferation compared to total CD3^+^ T cells in cultures of BMMC from MM patients (*N* = 10), with the highest expansion of T-cell subsets expressing LAG3. **B**. Treatment of BMMC from MM patients (*N* = 10) with clinical grade anti-PD1, anti-LAG3, or anti-OX40 enhanced proliferation of T cells in both CD4^+^ Th cells and CD8^+^ Tc cells, with the highest (*p < 0.05) increase with anti-LAG3 treatment. **C** Treatment of newly diagnosed (*N* = 6) or relapsed (*N* = 3) MM patients PBMC with clinical grade anti-LAG3 or anti-OX40 enhanced proliferation of CD3^+^ T cells, with the highest (*p < 0.05) increase with anti-LAG3 treatment, with or without MM lysates stimulation.

We first evaluated proliferation to low dose IL-2 in specific T-cell subsets in BMMC or PBMC from patients with newly diagnosed, relapsed, or relapsed/refractory MM using CFSE-based assays. Proliferation of CD3^+^ T cells expressing PD1, LAG3, OX40, or GITR was significantly (*p < 0.05) higher as compared to total CD3^+^ T cells in MM patient BMMC. In BMMC from patients (*N* = 10) with newly diagnosed, relapsed, or relapsed/refractory MM, T-cell subsets expressing the LAG3 immune checkpoint demonstrated the highest (*p < 0.05) proliferation (Fig. 1A; histograms, bar graph). We next assessed the impact of clinical grade immune modulators on T-cell proliferation within BMMC from patients with newly diagnosed, relapsed, or relapsed/refractory MM (*N* = 10). Overall, CD3^+^ T-cell proliferation was triggered by treatment with each clinical grade antibody (PD1, LAG3, OX40, GITR) compared to untreated control (Fig. 1B). Of note, a significant increase (*p < 0.05) in proliferation of CD4^+^ Th cells was induced by treatment with anti-PD1 or anti-LAG3, and increased (*p < 0.05) proliferation of CD8^+^ Tc cells after treatment with anti-LAG3 or anti-OX40. The highest proliferation in both CD4^+^ Th cells and CD8^+^ Tc cells was induced by anti-LAG3 treatment (histograms, bar graphs). Next, MM patient T-cell proliferation in response to tumor lysates from ten different MM cell lines, in the presence or absence of immune modulator, was examined. As shown in Fig. 1C, PBMC from patients with newly diagnosed (*N* = 6) or relapsed (*N* = 3) MM treated with anti-LAG3 had significantly (*p < 0.05) higher T-cell proliferation than with the other clinical grade immune modulators anti-PD1, anti-OX40, and anti-GITR, either in the presence or absence of MM lysate stimulation. In addition, BMMC from MM patients (*N* = 5) treated with anti-LAG3 had significantly higher (*p < 0.05) T-cell proliferation than with the other clinical grade immune modulators, upon stimulation with the mixture of ten different MM cell lines, either as irradiated whole cells or tumor lysates. The tumor lysates induced a greater T-cell response in MM patients’ BMMC (*N* = 5) than irradiated whole tumor cells, which was enhanced to a greater extent by checkpoint inhibitors (anti-LAG3 > anti-PD1) than by immune agonists (anti-OX40, anti-GITR) (Supplementary Fig. 1). Taken together, these data indicate the therapeutic potential of LAG3 blockade to effectively augment T-cell proliferation directed against MM.

### Decreased effector CD4^+^ Th cells, increased regulatory and immune suppressor cells, and upregulation of immune checkpoints in active MM patients compared to MGUS/SMM patients or healthy donors

**Fig. 2:**
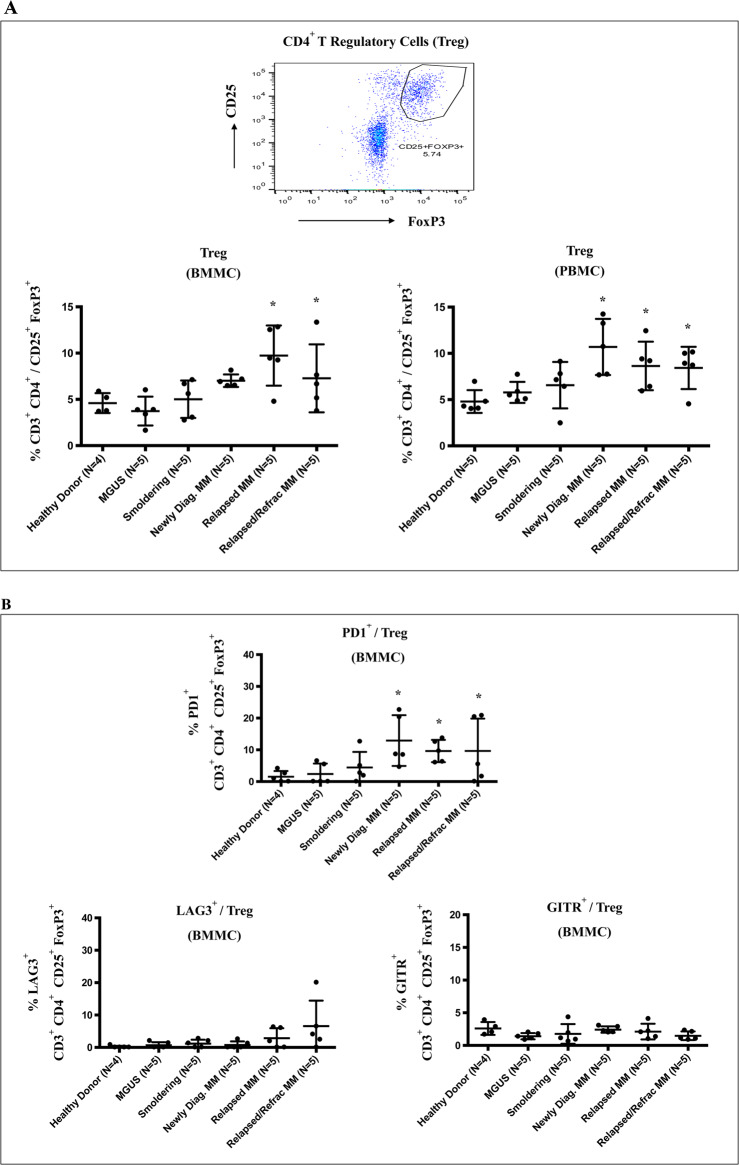
Characterization of regulatory T cells in BMMC or PBMC from patients with MGUS, SMM, or MM and healthy individuals. Freshly isolated BMMC or PBMC from patients with MGUS (BM: *N* = 5, PB: *N* = 5), SMM (BM: *N* = 5, PB: *N* = 5), newly diagnosed MM (BM: *N* = 5, PB: *N* = 5), relapsed MM (BM: *N* = 5, PB: *N* = 5), relapsed/refractory MM (BM: *N* = 5, PB: *N* = 5), and healthy donors (BM: *N* = 4, PB: *N* = 5) were evaluated for the frequency of regulatory T cells and their expression of immune checkpoints. **A** MM patients (newly diagnosed, relapsed, relapsed/refractory) had a significantly (*p < 0.05) higher CD4^+^ Treg (CD25^+^FOXP3^+^/CD3^+^CD4^+^) in BMMC and PBMC compared to MGUS patients, SMM patients or healthy individuals. **B** CD4^+^ Treg cells from active MM patients had significantly (*p < 0.05) higher PD1, but not LAG3 or GITR, expression in BMMC as compared to MGUS/SMM patients or healthy individuals.

**Fig. 3:**
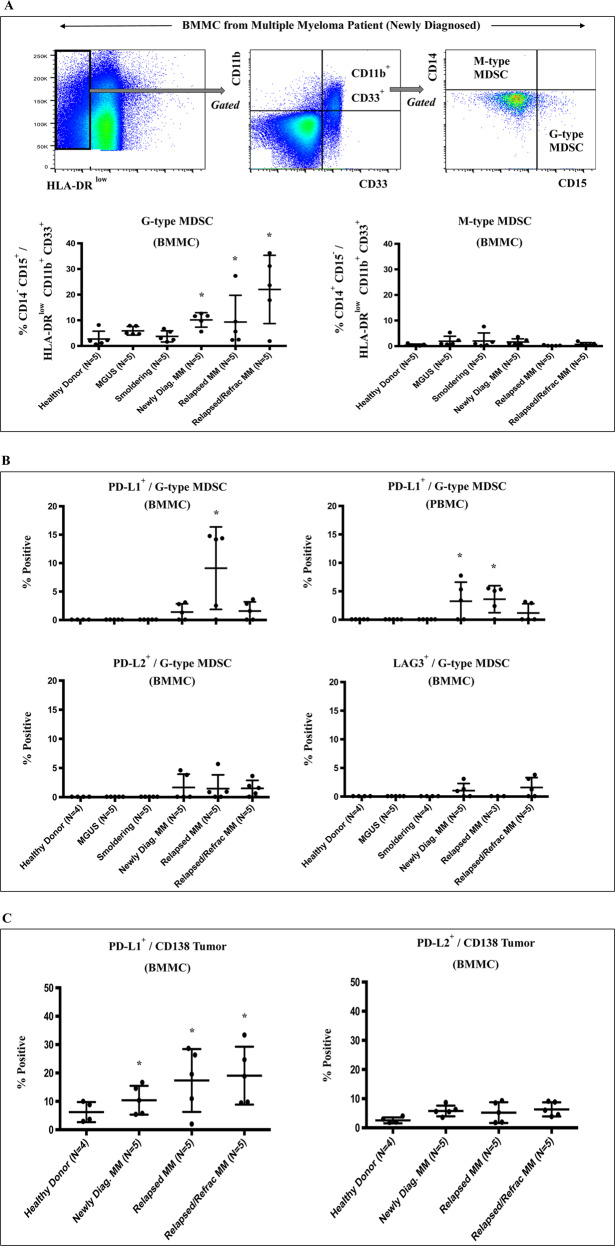
Characterization of MDSC and CD138^+^ MM cells in BMMC or PBMC from patients with MGUS, SMM, or MM and healthy donors. Freshly isolated BMMC or PBMC from patients with MGUS (BM: *N* = 5, PB: *N* = 5), SMM (BM: *N* = 5, PB: *N* = 5), newly diagnosed MM (BM: *N* = 5, PB: *N* = 5), relapsed MM (BM: *N* = 5, PB: *N* = 5), relapsed/refractory MM (BM: *N* = 5, PB: *N* = 5), and healthy donors (BM: *N* = 4, PB: *N* = 5) were evaluated for the frequency of MDSC or CD138^+^ MM cells and their expression of immune checkpoints. **A** MM patients BMMC had significantly (*p < 0.05) higher G-type MDSC (CD11b^+^ CD33^+^ HLA-DR^low/−^ CD14^−^ CD15^+^), but not M-type MDSC (CD11b^+^ CD33^+^ HLA-DR^low/−^ CD14^+^ CD15^−^), compared to MGUS/SMM patients or healthy individuals. **B** G-type MDSC from MM patients BMMC and PBMC expressed significantly (*p < 0.05) higher PD-L1, but not PD-L2 or LAG3, than MGUS and SMM patients or healthy individuals. **C** CD138^+^ tumor cells in MM patient BMMC have significantly (*p < 0.05) higher PD-L1, but not PD-L2, expression as compared to BMMC from healthy individuals.

We and others have characterized the impact of interactions among tumor, stromal, and accessory cells on MM cell growth, survival, and drug resistance [1–3]. We therefore next evaluated immune effector, regulatory/suppressor, and tumor cells for expression of key immune checkpoints using freshly isolated BMMC and PBMC from patients with MGUS, SMM, or MM, as well as normal healthy individuals. Compared to MGUS/SMM patients or healthy individuals, MM patients’ (newly diagnosed, relapsed, relapsed/refractory) BMMC and PBMC had significantly (*p < 0.05) decreased CD4^+^ Th, but not CD8^+^ Tc cells (data not shown), as well as increased CD4^+^ Treg (CD3^+^CD4^+^/FOXP3^+^CD25^+^) (Fig. 2A). Of note, PD1 was more highly expressed on CD4^+^ Treg within BMMC from MM patients than patients with MGUS/SMM or healthy individuals and demonstrated higher expression of PD1 than LAG3 or GITR (Fig. 2B). We next showed that G-type MDSC (CD11b^+^ CD33^+^ HLA-DR^low/−^ CD14^−^ CD15^+^), but not M-type MDSC (CD11b^+^ CD33^+^ HLA-DR^low/−^ CD14^+^ CD15^−^), are significantly (*p < 0.05) increased in BMMC of MM patients (highest in relapsed/refractory MM) compared to MGUS/SMM patients or healthy donors (Fig. 3A). Moreover, G-type MDSC in BMMC and PBMC of MM patients expressed significantly higher levels of PD-L1 than PD-L2 or LAG3 (Fig. 3B). Finally, CD138^+^ MM cells (newly diagnosed, relapsed, relapsed/refractory) had significantly (*p < 0.05) higher expression of PD-L1, but not PD-L2, than healthy donors (Fig. 3C). In addition, CD4^+^ Th cells and CD8^+^ Tc cells in MM patients had significantly higher PD1 expression compared to MGUS/SMM patients or healthy individuals (data not shown). Thus, these studies reveal heterogeneity in the proportion of immune cell subsets among patients with MGUS, SMM, or MM, and healthy individuals; decreased effector cells, increased Treg, and G-type MDSC, as well as upregulation of immune checkpoints on effector, regulatory, and CD138^+^ MM cells are observed in MM patients compared to patients with MGUS or SMM or healthy individuals.

### Higher intracellular than surface expression of immune checkpoints in MM patient BM

**Fig. 4:**
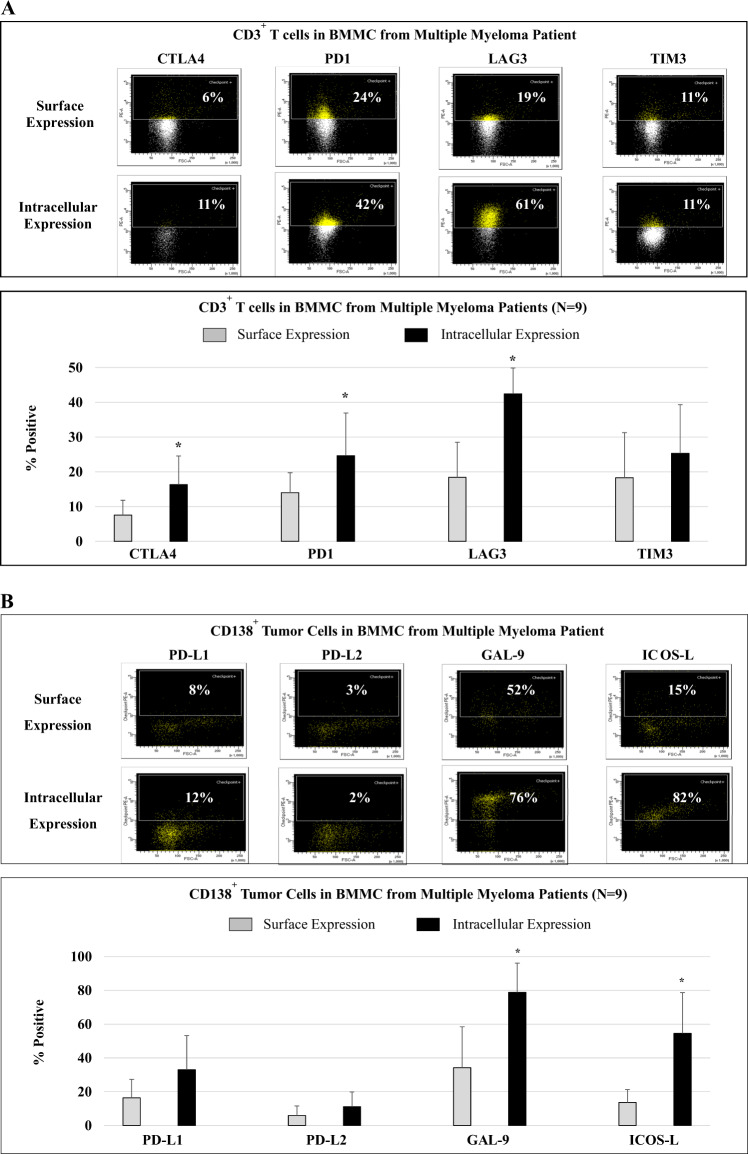
Distribution, location, and expression level of key immune checkpoints, activation, and costimulatory molecules in MM patients’ BMMC. Cell surface and intracellular expression of immune checkpoints were evaluated and compared in BMMC from MM patients (newly diagnosed, relapsed, relapsed/refractory; *N* = 9) using flow cytometry. **A** CD3^+^ T cells in BMMC from MM patients showed an increased expression of PD1 and LAG3 than CTLA4 or TIM3. Intracellular expression of CTLA4, PD1, and LAG3 was significantly (*p < 0.05) greater than corresponding cell surface expression levels. **B** Primary CD138^+^ patient MM cells showed increased cell surface expression of GAL-9/ICOS-L as compared with PD-L1/PD-L2, as well as significantly (*p < 0.05) higher intracellular expression of GAL-9 and ICOS-L than cell surface expression.

To better understand potential mechanisms of resistance to checkpoint inhibitor therapy in MM, we next examined the distribution and localization (cell surface vs. intracellular) of key immune checkpoints in BMMC from MM patients (*N* = 9). CD3^+^ T cells more highly expressed PD1 and LAG3 than CTLA4 and TIM3, with significantly (*p < 0.05) greater intracellular CTLA4, PD1, and LAG3 expression than cell surface levels (Fig. 4A; histograms, bar graph). In contrast, surface and intracellular expression levels of TIM3 were similar. On the CD138^+^ MM cells, GAL-9 and ICOS-L were more highly expressed than PD-L1 and PD-L2, with higher intracellular than cell surface expression of PD-L1, PD-L2, GAL-9, and ICOS-L (Fig. 4B; histograms, bar graph). These results support an extended treatment protocol with checkpoint inhibitors to overcome the high intracellular reservoir of immune checkpoints, and thereby overcome immunosuppression and improve outcome in MM.

### Induction of another checkpoint expression and Treg triggered by treatment with checkpoint inhibitor or immune agonist

**Fig. 5:**
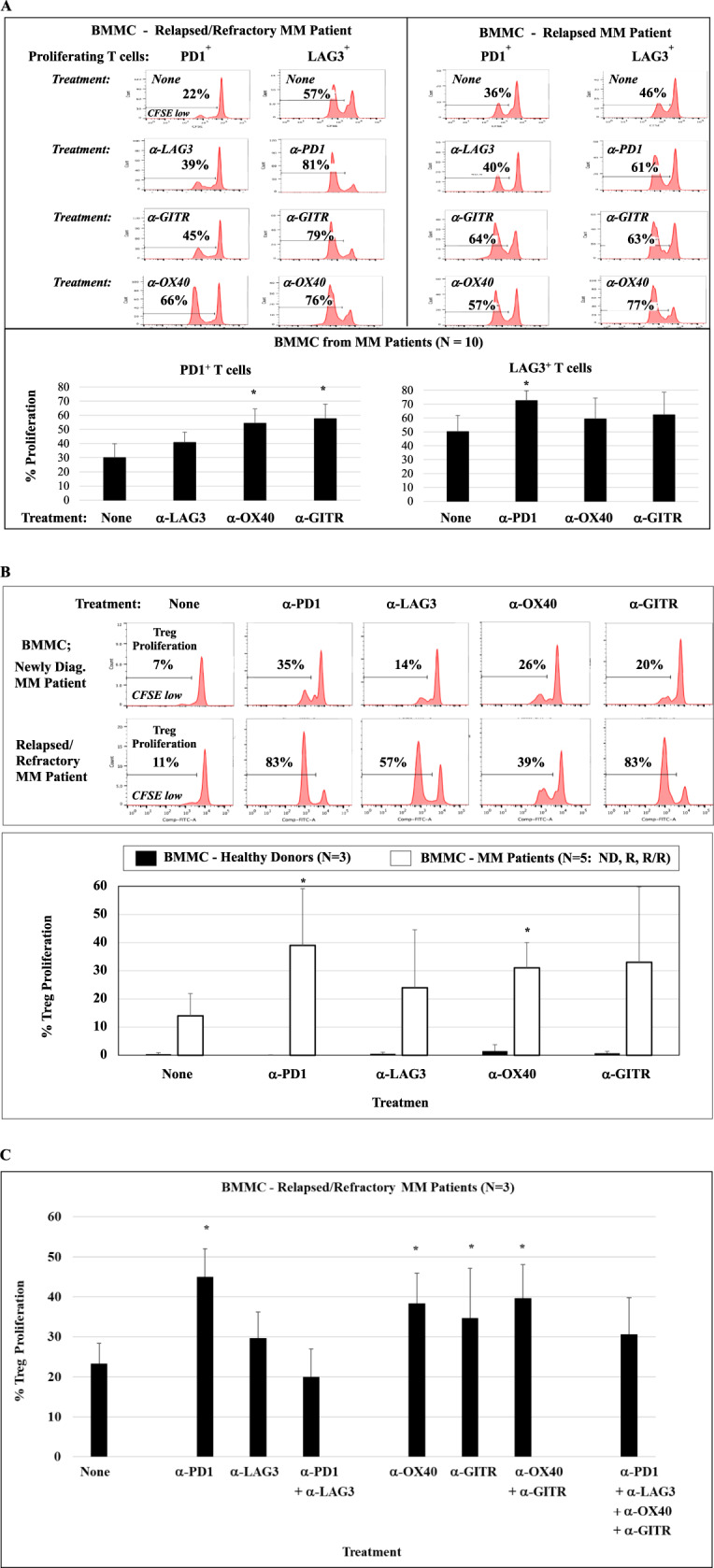
Impact of checkpoint inhibitor or immune agonist treatment on MM patients’ BMMC. BMMC from MM patients were treated with clinical grade checkpoint inhibitor or immune agonist in the presence of low level of IL-2 (20 units/ml) and examined for checkpoint expression and immune function. **A** Treatment of BMMC from MM patients (*N* = 10) with anti-PD1, anti-LAG3, anti-OX40, or anti-GITR increased expansion of T cells expressing another immune checkpoint. **B** Treatment of BMMC of MM patients (*N* = 5) induced CD4^+^ Treg proliferation, with the highest increase triggered by anti-PD1 (*p < 0.05) and the lowest by anti-LAG3. **C** Treatment of BMMC from MM patients (*N* = 3) with single agent anti-PD1, anti-OX40, or anti-GITR enhanced (*p < 0.05) proliferation of Treg, which was decreased by combination treatment with checkpoint inhibitor (α-PD1 + α-LAG3) compared to combination with immune agonist (α-OX40 + α-GITR) or single agent treatment.

To further elucidate potential mechanisms of resistance to checkpoint inhibitor or immune agonist therapy in MM patients, we next evaluated the effects of clinical grade modulators on effector and Treg cell subsets in tumor microenvironment. Importantly, treatment of MM patients’ (*N* = 10) BMMC with the specific mAb targeting PD1, LAG3, OX40, or GITR induced upregulation of PD1 and LAG3 expression on T cells (Fig. 5A; histograms, bar graph). Of note, anti-PD1 triggered proliferation of T cells expressing an alternative immune checkpoint to a greater extent than anti-LAG3 treatment. Moreover, checkpoint inhibitor or immune agonist treatment of MM patient’ (*N* = 5) BMMC increased Treg proliferation, with a significantly (*p < 0.05) high induction by anti-PD1 or anti-OX40 and the lowest induction by anti-LAG3 (Fig. 5B; histograms, bar graph). We also investigated the impact of single agent or combination modulator treatment on Treg expansion in the MM microenvironment (Fig. 5C). Treatment of MM patients’ (*N* = 3; relapsed/refractory) BMMC with single agent anti-PD1, anti-OX40, or anti-GITR triggered a significant (*p < 0.05) expansion of Treg. A decreased level of Treg proliferation was observed upon combination treatment with checkpoint inhibitors, which was not detected in combination treatment with immune agonists; among various combination treatments evaluated, the lowest level of Treg proliferation was noted with anti-PD1 plus anti-LAG3. These results indicate a potential mechanism of immune resistance to checkpoint therapy whereby treatment with a checkpoint inhibitor induces immune suppressive cells and nontargeted immune checkpoints in MM patients BMMC, suggesting the need for combination modulator treatment to overcome resistance to single agent immunotherapy approaches.

### Increased functional anti-MM activity of XBP1/CD138/CS1-specific CTL treated with anti-LAG3

**Fig. 6:**
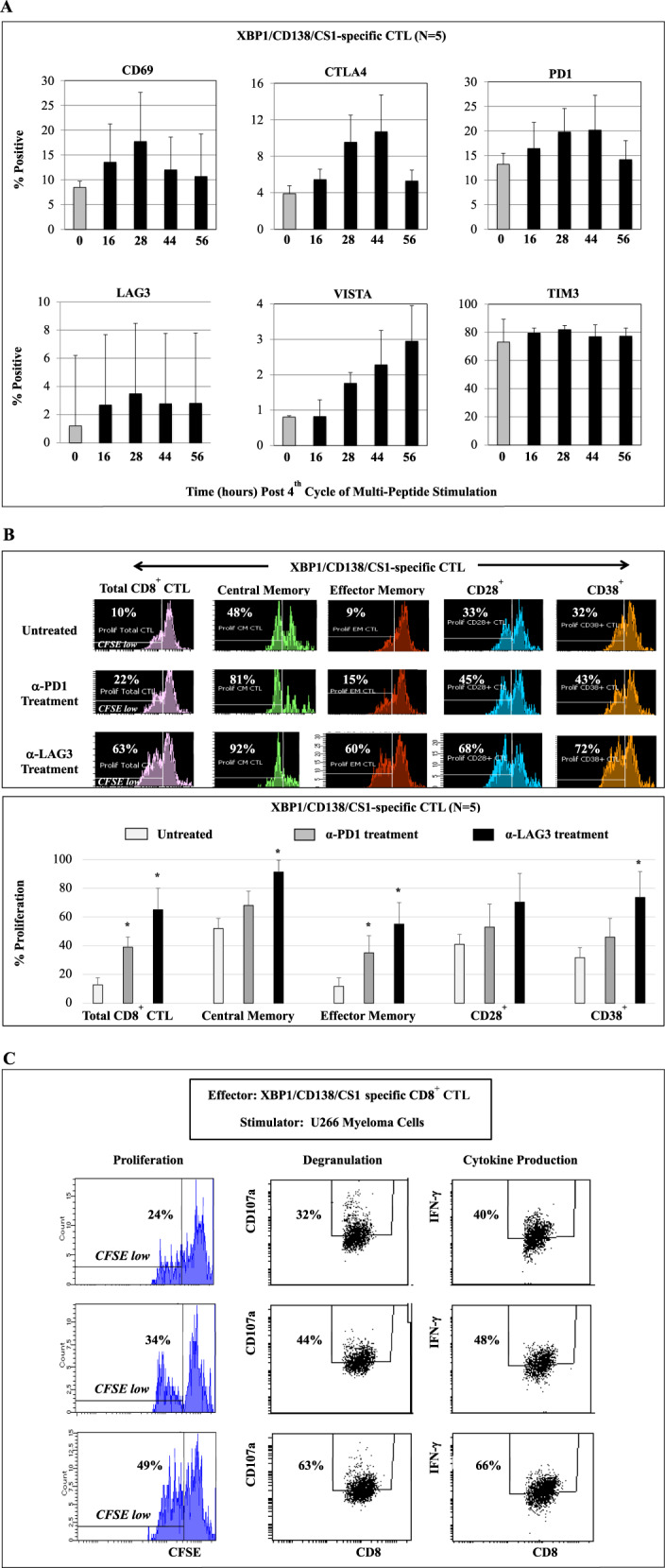
Impact of checkpoint inhibitor treatment on anti-MM activities of XBP1/CD138/CS1-specific CTL. HLA-A2-specific XBP1/CD138/CS1-specific CTL (*N* = 5) were generated by four cycles of weekly stimulation of CD3^+^ T cells with immunogenic XBP1/CD138/CS1 peptides and then examined for their phenotypic profile and functional activities against MM. **A** Upon fourth cycle of peptides stimulation, time-dependent T-cell activation (CD69) and checkpoint (CTLA4, PD1, LAG3, VISTA, TIM3) upregulation were detected on MM-specific CTL. **B**, **C** Immune checkpoint treatment enhanced (anti-LAG3 > anti-PD1) the poly-functional activities of antigen-specific T cells in response to HLA-A2-matched U266 MM cells. **B** Induced proliferation of total CD8^+^ CTL as well as central memory, effector memory, CD28^+^ and CD38^+^ CTL subsets. **C** Increased CD107a^+^ degranulation and IFN-γ production associated with a higher CD8^+^ CTL proliferation.

We next evaluated the functional significance of immune modulator therapy by examining its impact on anti-tumor activity of MM-specific CTL generated with HLA-A2 XBP1/CD138/CS1 peptides including heteroclitic XBP1 US_184-192_ (YISPWILAV), heteroclitic XBP1 SP_367-375_ (YLFPQLISV), native CD138_260-268_ (GLVGLIFAV), and native CS1_239-247_ (SLFVLGLFL), as described previously [14–17]. Phenotypic analyses after four cycles of weekly stimulation with peptides demonstrated time-dependent increased expression of CD69 activation marker and CTLA4, PD1, LAG3, and VISTA immune checkpoints on XBP1/CD138/CS1-specific CTL (*N* = 5) (Fig. 6A). In response to stimulation with HLA-A2 matched MM cells (U266), the central memory CD8^+^ T-cell subset displayed the highest proliferation (48%). Importantly, the XBP1/CD138/CS1-CTL treated with clinical grade anti-LAG3 or anti-PD1 increased (α-LAG3 > α-PD1) a significant (*p < 0.05) proliferation of total CD8^+^ T cells as well as CM, EM, CD28^+^, and CD38^+^ CTL subsets (Fig. 6B; histograms, bar graph [*N* = 5]). The treatment with each checkpoint inhibitor also increased anti-tumor activities of XBP1/CD138/CS1-CTL against MM cells, evidenced by increased CD107a degranulation and IFN-γ production, with the highest anti-tumor activities induced by anti-LAG3 treatment (Fig. 6C). These results further support the ability of LAG3 blockade to augment anti-MM immune responses including antigen-specific memory CTL, their cytotoxic activities, and Th1 cytokine production against tumor.

### Surface and intracellular expression of LAG3 ligands, GAL-3 and HLA-DP/DQ/DR, in CD138^+^ cells in MM patient BMMC, and induction of specific CD8^+^ Tc proliferation by blocking GAL-3

**Fig. 7:**
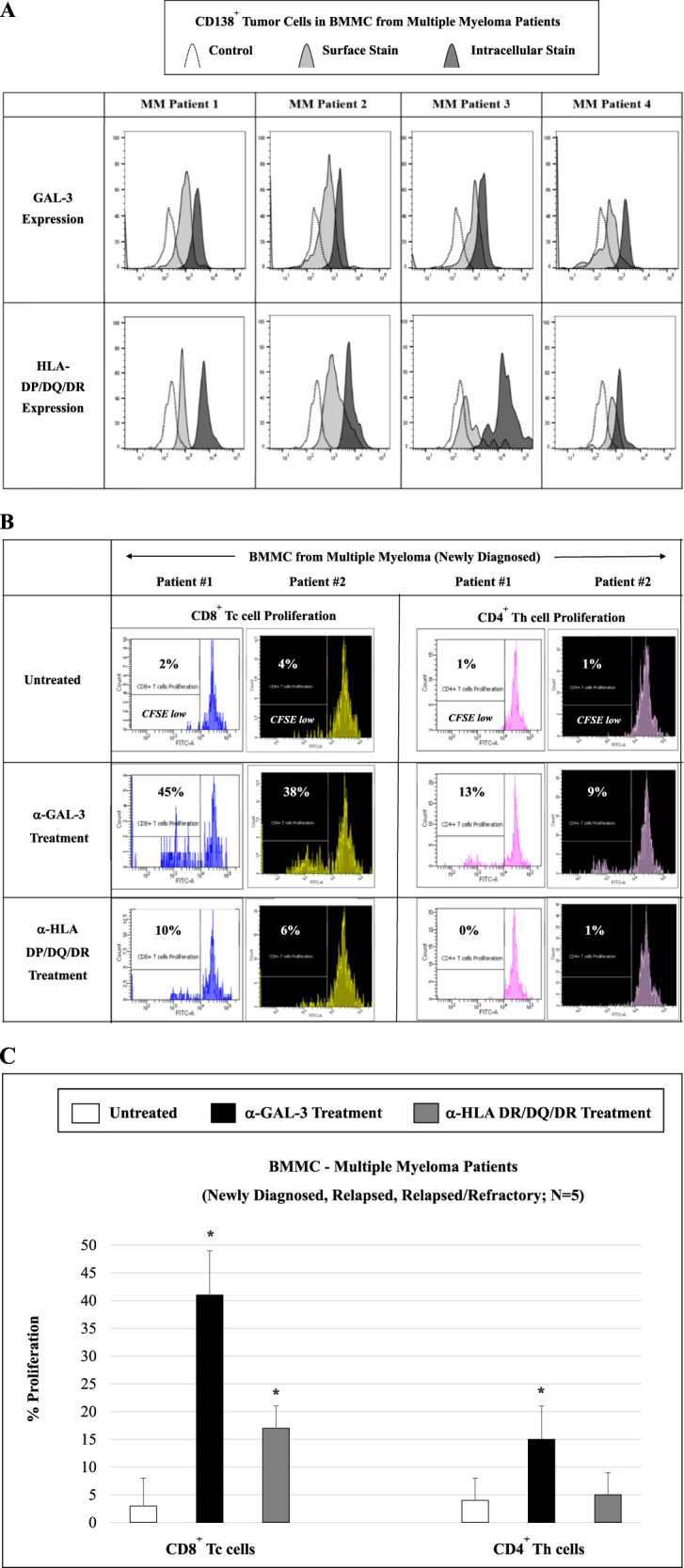
Expression and role of LAG3 ligands, GAL-3 and HLA-DP/DQ/DR, on CD138^+^ cells in BMMC or PBMC from MM patients. Phenotype and functional characterization of LAG3 ligands, GAL-3 and HLA-DP/DQ/DR, were evaluated in MM patients’ BMMC by flow cytometry. **A** GAL-3 and HLA-DP/DQ/DR were both expressed (intracellular > cell surface) in CD138^+^ tumor cells from MM patients (*N* = 4). **B** Treatment of MM patient BMMC with anti-GAL-3, but not with anti-HLA-DP/DQ/DR, increased proliferation of T cells (CD8^+^ Tc > CD4^+^ Th) in BMMC from MM patients (Patient #1, Patient #2). **C** GAL-3 blockade induced an increased (*p < 0.05) proliferation of both CD8^+^ Tc and CD4^+^ Th cells (CD8^+^ > CD4^+^) in BMMC from MM patients (*N* = 5).

Having shown the functional significance of the LAG3 immune checkpoint in MM, we next assessed its ligands GAL-3 and HLA-DP/DQ/DR. The expression and role of each of LAG3 ligand was analyzed in BMMC from MM patients (*N* = 4; newly diagnosed, relapsed, relapsed refractory). CD138^+^ MM cells demonstrated the expression of both GAL-3 and HLA-DP/DQ/DR, with greater expression evidenced by higher median fluorescence intensity intracellularly than on the cell surface (Fig. 7A). We next examined the functional significance of GAL-3 and HLA-DP/DQ/DR blockade in MM. Treatment of newly diagnosed MM patient BMMC with anti-GAL-3, but not with anti-HLA-DP/DQ/DR, induced proliferation of T cells, including CD8^+^ Tc cells (45%: Patient #1, 38%: Patient #2) and CD4^+^ Th cells (13%: Patient #1, 9%: Patient #2) (Fig. 7B). Additional analyses of BMMC from MM patients (newly diagnosed, relapsed, relapsed/refractory; *N* = 5) showed a significant (*p < 0.05) increase in proliferation of CD8^+^ Tc and CD4^+^ Th cells (CD8^+^ > CD4^+^) triggered by GAL-3 blockade (Fig. 7C). These results indicate the potential benefit of GAL-3 blockade in MM patients to induce T cells specific responses with a high level of CD8^+^ T cells proliferation.

### Induction of MM-specific CD8^+^ T-cell proliferation and anti-tumor activities by blocking GAL-3 on MM cells

**Fig. 8:**
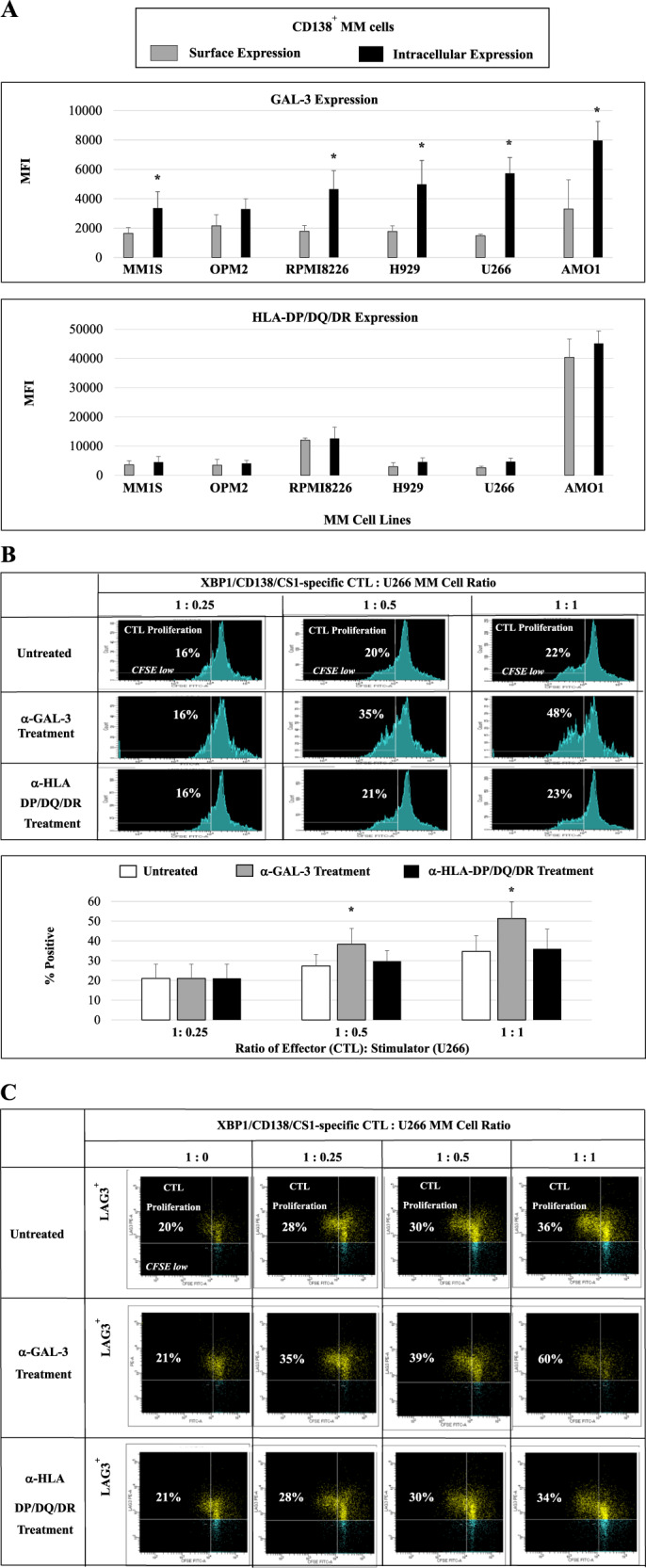
Impact of inhibition of LAG3 ligands on proliferation and antitumor activities of MM-specific CTL. The role of the LAG3 ligands was analyzed using XBP1/CD138/CS1-specific CD8^+^ CTL against MM cells. **A** GAL-3 and HLA-DP/DQ/DR are both expressed in CD138^+^ tumor cells of MM cell lines (*N* = 3), with higher (*p < 0.05) intracellular than cell surface expression of GAL-3. **B** HLA-A2^+^ XBP1/CD138/CS1-specific CTL (*N* = 5) demonstrated increased MM-specific CD8^+^ CTL proliferation in response to HLA-A2^+^ U266 MM cells treated with anti-GAL-3, but not with anti-HLA-DP/DQ/DR, in a dose (U266 cells)-dependent manner (XBP1/CD138/CS1-CTL: MM cells = 1:1 > 1:0.5 > 1:0.25). **C** Proliferation of LAG^+^ cells was triggered in XBP1/CD138/CS1-specific CTL by stimulation with U266 treated with anti-GAL-3, but not with anti-HLA-DP/DQ/DR, in a dose (U266)-dependent manner (XBP1/CD138/CS1-CTL: MM cells = 1:1 > 1:0.5 > 1:0.25 > 1:0).

Finally, we examined the impact of inhibiting LAG3 ligand on MM-specific CTL activities. We first extended our analysis of GAL-3 and HLA-DP/DQ/DR to MM cell lines (MM1S, OPM2, RPMI8226, H929, U266, AMO1). Overall, a higher HLA-DP/DQ/DR surface and intracellular expression was detected than GAL-3 expression, whereas GAL-3 displayed a greater (*p < 0.05) level of intracellular than cell surface expression (*N* = 3) (Fig. 8A). We further examined the functional impact of GAL-3 or HLA-DP/DQ/DR blockade on the specific proliferation and anti-tumor activities of MM-specific CD8^+^ CTL against MM cells in an HLA-A2-specific manner. As shown in Fig. 8B, proliferation of HLA-A2 XBP1/CD138/CS1-specific CTL was enhanced in response to HLA-A2^+^ U266 MM cells upon the treatment with anti-GAL-3, but not with anti-HLA-DP/DQ/DR, in an effector (CTL):target (MM cells)-dependent manner (1:1 > 1:0.5 > 1:0.25) (histograms, bar graphs [*N* = 5]). Importantly, the specific blockade of GAL-3 in MM cells further increased proliferation of LAG3 expressing XBP1/CD138/CS1-CTL (Fig. 8C), suggesting an alternative escape mechanism after anti-GAL-3 therapy in MM patients. Taken together, these results identify the functional relevance of blocking GAL-3 on MM cells as a means to enhance effector T-cell activities, and also provide the rationale for targeting GAL-3 (on MM tumor cells) in combination with LAG3 (on effector T cells) to further enhance MM-specific immune responses and anti-tumor activities.

## Discussion

Understanding the biologic and immune sequelae of tumor cell interaction with accessory and immune cells in the tumor microenvironment is crucial for the development of successful cancer immunotherapies. Effective therapeutic strategies in MM may not only target tumor and tumor-promoting accessory cells, but also abrogate mechanisms mediating immunosuppression in the BM milieu [21–23]. Previously, our group has delineated the role of accessory cells (MDSC, plasmacytoid dendritic cells, Treg, osteoclasts) in promoting tumor cell growth and survival and drug resistance, as well as conferring immunosuppression in MM [24–26]. In the current study, we further characterized the distribution, location, and expression levels of immune checkpoints not only on effector T cells, but also on MM cells and immune regulatory/suppressor cells in the BM and PB of patients with MM (newly diagnosed, relapsed, relapsed/refractory), premalignant diseases (MGUS, SMM), and healthy individuals. Our analyses revealed key differences in the frequency of cellular subsets (immune effector, regulatory/suppressor vs. tumor cells) and expression of immune checkpoints/agonists in patients with active MM compared to MGUS/SMM and healthy donors.

Since effective immunotherapy depends upon robust effector T-cell function [27–29], we first defined the presence and function of endogenous T-cell subsets in MM patient BM and PB. Although proliferating CD3^+^ T cells in the presence of IL-2 or MM cell lysates expressed multiple immune modulators in these studies, the immune checkpoint LAG3 was most highly expressed on both proliferating CD4^+^ Th and CD8^+^ Tc cells, and anti-LAG3 treatment most significantly enhanced their MM-specific immune responses. Our studies further demonstrated decreased effector CD4^+^ Th cells, increased Treg and G-type MDSC, as well as upregulation of immune checkpoints on both effector/regulatory cells and patients CD138^+^ tumor cells in MM, compared to patients with MGUS and SMM or healthy individuals. Of immune modulators profiled, LAG3 expression and impact of anti-LAG3 treatment was low on G-type MDSC and Tregs, suggesting that it will not enhance immunosuppression conferred by these accessory cells in the BM milieu. In evaluation of XBP1/CD138/CS1 peptides-specific CTL with anti-MM activity, we confirmed that anti-LAG3 treatment induced enhanced proliferation of both CM and EM memory CTL subsets and their functional anti-MM activities including cytotoxicity and Th1-type cytokine production. Importantly, we also identified GAL-3, the ligand for LAG3, to be robustly expressed on CD138^+^ MM cells, and confirmed that anti-GAL-3 treatment can similarly augment immune responses against MM cells in patient BM, as well as XBP1/CD138/CS1 antigen-specific CTL. These studies identify and validate the potential blockade of LAG3/GAL-3 to enhance anti-tumor immune responses in MM.

Checkpoint blockade is a revolutionary cancer immunotherapy; however, a large proportion (70–80%) of checkpoint inhibitor-treated cancer patients do not benefit due to either intrinsic or acquired resistance [30–33]. Multiple factors contribute to checkpoint blockade resistance including a lack of antigen-specific immune responses and/or impaired infiltration of effector T cells to tumor sites [34–39]. An important goal of our studies was to better elucidate potential mechanisms whereby immune inhibitory receptors and ligands regulate innate and adaptive immunity in MM, and specifically delineate potential mechanisms of resistance to checkpoint blockade in MM. We demonstrated a direct beneficial impact of checkpoint inhibitor treatment on T-cell functional activity in BM cells from MM patients. Specifically, checkpoint inhibitor (especially anti-LAG3) treatment significantly increased T-cell responses in BMMC/PBMC from MM patients compared with healthy donors. Along with identifying the potential functional role of checkpoint inhibitors, we also examined the impact of immune agonists such as OX40 (CD134) and GITR (CD357) to activate costimulatory molecules on effector cells and thereby enhance their immune responses [36, 37]. In both MM patient BMMC and PBMC, immune agonist treatment enhanced immune responses and T-cell proliferation. Importantly, our finding of higher intracellular than cell surface checkpoint expression on CD3^+^ T cells and CD138^+^ MM cells in patient BMMC suggests that high intracellular levels of checkpoints may provide a continuous source of checkpoint molecules for translocation to the cell surface, thereby maintaining ongoing checkpoint-driven immune resistance in MM. Moreover, our studies show that treatment of MM patient BMMC with one checkpoint inhibitor can upregulate expression of another checkpoint, as well as expansion of regulatory and suppressor cells, in addition to effector CD3^+^ T cells. Taken together, these data identify alternative mechanisms of immune resistance induced by checkpoint inhibitors and immune agonists in MM.

Among the clinical grade checkpoint inhibitors and immune agonists evaluated in these studies, anti-PD1 treatment induced the highest level of CD4^+^ Treg expansion and upregulation of other immune checkpoints. Importantly, anti-LAG3 treatment induced the most robust effector T-cell proliferation, while inducing the lowest level of induction of other checkpoints and Treg expansion. In addition, we detected a higher intracellular expression of LAG3 compared to PD1 in MM patient (*N* = 5) BMMC. Based on these findings, we suggest that LAG3 blockade in MM may be more effective than PD1 blockade, with a lower induction of alternative checkpoint molecules and a higher induction of effector T-cell proliferation and response. These results are of particular relevance, given recent toxicity concerns observed when combining pembrolizumab (anti-PD1) with immunomodulatory drugs lenalidomide or pomalidomide or with daratumumab (anti-CD38) in recent clinical trials for relapsed MM patients [40–42]. Considering the robust LAG3 expression in the tumor microenvironment and its correlation with poor prognosis in MM and other cancers [23, 43–46], targeting the LAG3-specific inhibitory pathway may enhance anti-MM immunity and have a more favorable therapeutic index. Importantly, among the clinical grade checkpoint inhibitors and immune agonists evaluated in these studies, anti-LAG3 treatment significantly enhanced the proliferation of MM-specific effector cells and their functional activities in response to MM. Based on these results, we propose that anti-LAG3 may effectively overcome immunosuppression in the tumor microenvironment and be used, alone or in combination with other immune therapies such as MM-specific vaccination, to enhance generation and maintenance of antigen-specific memory CTL function against tumor.

Characterization of inhibitory checkpoint ligands as well as mechanisms of their interaction and consequent effector T-cell suppression are critical to better design clinical studies and achieve long-term anti-tumor immunity in MM patients. Pharmacological blockade of PD1 or PD-L1 has been at the forefront of immunotherapy for various cancers, as it reinvigorates exhausted T cells in the tumor microenvironment, thereby facilitating robust anti-tumor immune responses. However, up to 50% of patients with PD-L1 positive tumors show acquired resistance or relapse after an initial response to PD1/PD-L1 blockade [35, 47–49], highlighting the need to target alternative pathways of inhibitory checkpoint receptor/ligand interaction to improve clinical outcomes. To address this concern and in the context of our promising anti-LAG3 data in MM, we went on to evaluate expression of LAG3 ligands, GAL-3 and HLA-DP/DQ/DR, on CD138^+^ MM cells in patient BMMC and MM cell lines, identifying them as promising therapeutic targets. We found that blockade of GAL-3, but not HLA-DP/DQ/DR, enhanced proliferation of both CD4^+^ Th and CD8^+^ Tc cell subsets in BMMC from MM patients, independent of the cell surface or intracellular expression levels of the two respective ligands on primary CD138^+^ MM cells. Thus, we propose that checkpoint ligands expression level itself might not be the only factor, which influence effector T-cell function and proliferation. The expression level and specificity/affinity between checkpoint receptor (LAG3) on the patients’ T cells and the checkpoint ligands (GAL-3, HLA-DP/DQ/DR) on patients’ tumor cells are a critical consideration impacting the functional sequelae of their interaction. Recently, Kundapura and Ramagopal demonstrated that the CC′ loop of IgV domains of the immune checkpoint receptors, a loop which is distinct from CDRs of antibodies, plays a pivotal role in receptor: ligand affinity modulation [50]. They proposed that a ~5 amino acid residue long CC′ loop in a ~120 residue protein makes a significant number of hydrophobic and polar interactions with its cognate checkpoint ligand and suggested that the CC′ loop might be a hotspot for checkpoint receptor modification that enhance their affinity for ligand interaction. In addition, we propose that the interaction between receptor and ligand can be influenced by the unique T-cell receptor repertoire of each individual, resulting in variable levels or profiles of T-cell or CTL functional responses and proliferation. Taken together, our results in MM are consistent with previous reports on the role of GAL-3 as a key regulator of cell adhesion and inflammation in cancer [51–56]; it negatively regulates T-cell function and proliferation through interaction with LAG3, especially on CD8^+^ CTL, possibly by reducing the affinity of the T-cell receptor and its internalization. Importantly, we observed increased MM-specific CD8^+^ Tc cells expansion and selective anti-MM immune activities after anti-GAL-3 treatment of both MM patient BMMC and XBP1/CD138/CS1-specific CTL. These findings further indicate the potential role for LAG3 and/or GAL-3 inhibition, alone and with XBP1/CD138/CS1 peptide vaccination, to augment MM-specific memory CD8^+^ CTL anti-tumor activities against MM.

In summary, we have identified a key immunosuppressive role for LAG3 and its ligand GAL-3 in regulating innate and adaptive immunity in MM and provide the rationale for targeting LAG3/GAL-3, alone and in combination immunotherapeutic approaches, to improve patient outcome in MM.

## Supplementary information

Supplemental Figure 1
