# Supplementary material for: Targeting LAG3/GAL-3 to overcome immunosuppression and enhance anti-tumor immune responses in multiple myeloma
Source: Leukemia. 2021 Jul 21;36(1):138–54. doi: 10.1038/s41375-021-01301-6 (PMC8727303; doi:10.1038/s41375-021-01301-6)
Supplement: Supplementary file 1 — Supplemental Figure 1 [file 41375_2021_1301_MOESM1_ESM.docx]

**Supplemental Figure 1**

**Irradiated Tumor Cells**

**Tumor Lysates**

**Multiple Myeloma Patients’ (N=5) BMMC Stimulation**


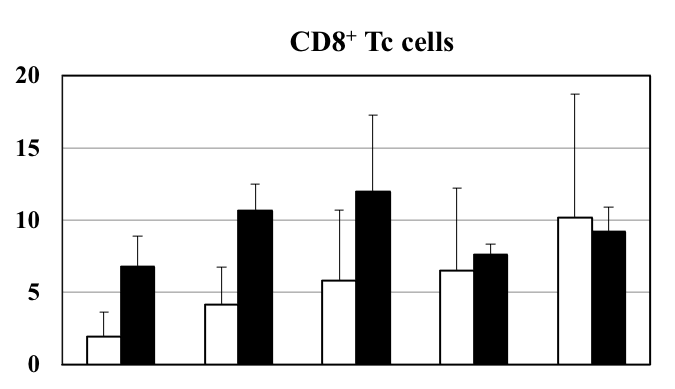


**Treatment**

**None α-PD1 α-LAG3 α-OX40 α-GITR**


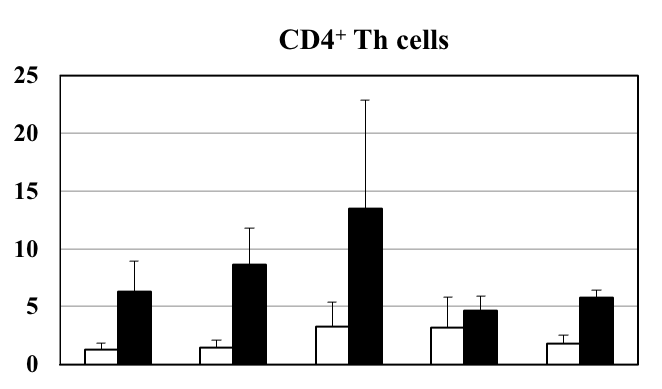


**None α-PD1 α-LAG3 α-OX40 α-GITR**


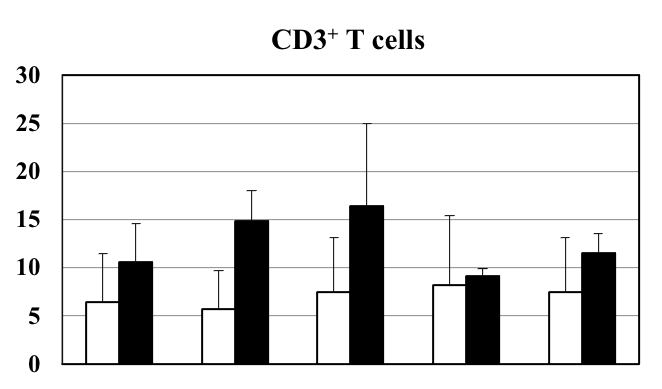


**% Proliferation**

**None α-PD1 α-LAG3 α-OX40 α-GITR**

*****

*****

*****

*****

*****

*****

**Treatment**
